# Uptake of human papilloma virus vaccine and its determinants among females in East Africa: a systematic review and meta-analysis

**DOI:** 10.1186/s12889-024-18141-5

**Published:** 2024-03-18

**Authors:** Muluken Chanie Agimas, Dagnew Getnet Adugna, Nebiyu Mekonnen Derseh, Amare Kassaw, Yohannes Tesfahun Kassie, Hailemichael Kindie Abate, Chilot Kassa Mekonnen

**Affiliations:** 1 Department of Epidemiology and Biostatistics, Institute of public health, College of Medicine and Health Science, University of Gondar, Gondar, Ethiopia; 2 Department of Human Anatomy, School of Medicine, College of Medicine and Health Science, University of Gondar, Gondar, Ethiopia; 3 Department of pediatric health nursing, college health science, Debre Tabor University, Debre Tabor, Ethiopia; 4 Department of Emergency and critical care nursing, college of health science, Debre Tabor University, Debre Tabor, Ethiopia; 5 Department of medical nursing, school of Nursing, College of Medicine and health science, University of Gondar, Gondar, Ethiopia

**Keywords:** HPV vaccine uptake, Determinants, East Africa

## Abstract

**Introduction:**

Cervical cancer is the most common malignant tumor among women. It is the main cause of death among women in sub-Saharan African countries. Particularly, the incidence and mortality rates are highest in East Africa. Even though the burden of human papilloma virus-related cervical cancer is high in East Africa, there is no conclusive evidence about the prevalence of human papilloma virus vaccine uptake and its predictors.

**Objective:**

To assess the pooled prevalence of human papilloma virus vaccine uptake and its determinants in East Africa.

**Method:**

Eligible articles were searched on PubMed, Embase, Scopus, Cochrane Library, Google Scholar, and Google. Those articles incorporating the outcome of interest, both analytical and descriptive study designs, and published or unpublished articles at any time were included. Keywords and Medical Subjects Heading terms or synonyms of human papilloma virus vaccine and Boolean operators were used to retrieve the articles. To assure the quality of articles, Joana Brigg’s Institute critical appraisal checklist for cross-sectional studies was used. Sensitivity analysis was conducted to assess the heterogeneity among the studies, and a random effect model was used to analyze the pooled effect size.

**Result:**

A total of 29 articles were included, and the pooled prevalence of HPV vaccine uptake in East Africa was 35% (95% CI: 26–45%). Good knowledge (OR = 1.6, 95%CI; 1.43–1.8), positive attitude (OR = 2.54, 95% CI; 2.13–3.03), ever heard about HPV vaccine (OR = 1.41, 95% CI; 1.03–1.94), mother educational status above college (OR = 1.84, 95%CI; 1.03–3.31), middle wealth index (OR = 1.33, 95%CI; 1.04–1.7), ≥ 9 family size (OR = 0.76, 95%CI; 0.68–0.98), availability of promotion (OR = 2.53, 95%CI: 1.51–4.26), availability of adequate vaccine (OR = 4.84, 95%CI; 2.9–8.08), outreach vaccination practice (OR = 1.47, 95%CI; 1.02–2.12) and family support (OR = 4.3, 95% CI; 2.98–6.21) were the significant factors for the uptake of human papilloma virus vaccine.

**Conclusion:**

As compared to the global strategic plan, the pooled prevalence of HPV vaccine uptake in east Africa was low. The uptake of the HPV vaccine was higher among adolescents than youths. Knowledge about the HPV vaccine, attitude towards the HPV vaccine, ever hearing about the HPV vaccine, residence, mother’s educational status, mother’s occupational status, wealth index, and family size were the significant determinants of HPV vaccine uptake. Therefore, we recommend focusing on awareness creation and behavioral change to expand the uptake of vaccines in East Africa.

**Supplementary Information:**

The online version contains supplementary material available at 10.1186/s12889-024-18141-5.

## Introduction

Cervical cancer is the fourth most common malignant tumor among women, and globally, about 604,127 new cases and 34,1831 deaths occurred in the year 2020 [1]. Of the total global deaths, about 90% occurred in low- and middle-income countries [2]. In developing countries, cervical cancer accounts for about 12% of all female cancers [3]. In Africa, it is the second form of cancer, with an incidence of 117,316 cases, and the total number of deaths by the year 2020 was 76,745 [4]. It is also the most common cause of death among women in sub-Saharan African countries [5]. East African countries contribute the highest numbers of incidence and death rates, at 27.6 and 42.7 per 100,000, respectively [6].

The major responsible cause of cervical cancer is the human papilloma virus. Human papilloma virus (HPV) 16 and 18 account for about 70% of all forms of cervical cancer [7]. Of all forms of HPV infection, about 10% advanced to precancerous lesions [8, 9]. Globally, about 11–12% of apparently healthy women have lived with HPV [10]. The incidence, prevalence, and mortality of cervical cancer are significantly reduced by the HPV vaccine, which can reduce cervical cancer-related death by 80% [11, 12]. According to the World Health Organization’s recommendation, vaccinating girls aged 9–14 is extremely effective when it takes place before sexual initiation and before HPV infection [13]. Of the three HPV vaccines, two of them, such as Gardasil and Cervarix, are accessible globally to prevent HPV-16 and HPV-18 strains [8]. Even though significant achievements are observed around the globe, low-income countries have encountered the difficulty of getting the HPV vaccine [14, 15]. In spite of the fact that the World Health Organization (WHO) recommends the introduction of the HPV vaccine for low-income countries, there is unequal distribution of the HPV vaccine across the countries because of differences in socioeconomics, culture, and knowledge about the HPV vaccine uptake [16]. According to the WHO report, the coverage of the HPV vaccine is higher in high-income countries (79%) than in upper-middle-income countries (58%), middle-income countries (26%), and low-income countries (21%) [17]. As the evidence showed, educational status [18], older age [19–21], ethnicity [18, 19, 22] and medium social economic status [20, 21] were the determinants of HPV vaccine uptake. Even though the burden of HPV-related cervical cancer is high, there is no conclusive and national-level evidence about the prevalence of HPV vaccine uptake and its determinants in East Africa. Therefore, this study was aimed at assessing the pooled prevalence of HPV vaccine uptake and its determinants among females in East African countries using a systematic review and meta-analysis.

## Methods

### Searching strategy

All the important published and unpublished papers were searched on PubMed, Embase, Scopus, Cochrane Library, Google Scholar, and Google, as well as the published article’s reference list, from March 20, 2023, to May 23, 2023. Especially articles from manual searches and published articles’ reference lists were searched for an extended period of time (2 months) to access all possible illegible articles exhaustively or to avoid the risk of missing the articles. The searching strategy was guided by the Preferred Reporting Items for Systematic Review and Meta-Analysis (PRISMA) guideline [23]. The searching mechanism was also established using Medical Subject Heading (MeSH) terms by combining the key terms of the title. Content experts were advised to obtain more papers or to reduce the risk of missing them. The seven independent reviewers, namely MCA, AK, NMD, DGA, HKA, CK, and YTK, participated in searching, and discussion was the solution to the disagreement during searching. This systematic review and meta-analysis was conducted by extracting studies in the context of the prevalence of HPV vaccine utilization using the following key entry terms: (uptake) OR (practice) AND (human papilloma virus vaccine) OR (HPV vaccine) AND (determinants) OR (predictors) OR (associated factors) AND (east Africa) (Table 1).

**Table 1:** a search strategy for the uptake of HPV vaccine among females in East Africa, 2023

MeSH Heading	Synonyms	Entry terms	Combination	Numbers Of article	Last date of searching	Electronic Databases & webs
Uptake of Human papillomavirus vaccine	Practice of the Human papillomavirus vaccine,	Uptake, practice, Human papillomavirus vaccine, HPV vaccine, determinants, predictors, associated factors, east Africa.	(Uptake) OR (practice) AND (Human papillomavirus vaccine) OR (HPV vaccine) AND (determinants) OR (predictors) OR (associated factors) AND (east Africa).	18,551	March 20, 2023	Medline/PubMed
			(Uptake) OR (practice) AND (Human papillomavirus vaccine) OR (HPV vaccine) AND (determinants) OR (predictors) OR (associated factors) AND (east Africa).	46	March 21, 2023	EMBASE
			(Uptake) OR (practice) AND (Human papillomavirus vaccine) OR (HPV vaccine) AND (determinants) OR (predictors) OR (associated factors) AND (east Africa).	3285	March 22, 2023	SCOPUS
			(Uptake) OR (practice) AND (Human papillomavirus vaccine) OR (HPV vaccine) AND (determinants) OR (predictors) OR (associated factors) AND (east Africa).	197	March 23, 2023	Cochrane Library
			(Uptake) OR (practice) AND (Human papillomavirus vaccine) OR (HPV vaccine) AND (determinants) OR (predictors) OR (associated factors) AND (east Africa).	18,300	March 24/2023	Google Scholar
				84	March 25/2023 to April 23/2023 April 24/2023 to May 23/2023	Google (Manually) Reference lists from the published articles

### Eligibility criteria

The reviewer and authors screened all the papers’ titles, abstracts, and full texts. Those articles incorporating the outcome of interest, namely the prevalence of HPV vaccine uptake or the determinants of the HPV vaccine, studies that conducted both analytical and descriptive study designs, and published or unpublished articles at any time were included in this systematic review and meta-analysis. Additionally, articles that did not have a full text and were published in other languages than English were also excluded.

### Screening and study selection

After searching the articles using keywords, MeSH terms, and Boolean operators in the websites and electronic databases, the articles were exported to Endnote X9 for further intensive screening. Duplicated articles were removed before the screening started. Then the PDF file was attached to endnote X9 for the rest of the articles. Finally, all articles in the endnote library were screened for eligibility using title, abstract, and full text. Those articles that did not incorporate the outcome of interest were removed, while articles that reported either HPV vaccine uptake prevalence or determinants of HPV vaccine uptake were kept for data extraction.

### Outcome of the study

The HPV vaccine uptake was an outcome of the current systematic review and meta-analysis, which was measured as yes for those who took the HPV vaccine and as no for those who did not take the HPV vaccine.

### Population

Females those were adolescents and youths.

### Data extraction

Next to the exhaustive data searching and screening, eligible articles were extracted using a data extraction format that includes the name of the first author, year of publication, year of data collection, country, study design, sample size, numbers of HPV vaccine uptake, the proportion of HPV vaccine uptake, standard error of the proportion, odds ratio, lower confidence interval of the odds ratio (OR), upper confidence interval (CI) of the OR, standard error of the OR, data collection tool, and quality score. Data extraction for the selected articles was also conducted by seven independent reviewers and authors, and the disagreement was resolved by the discussion. Finally, the extracted data were exported to STATA software version 14 for further analysis of the pooled effect size.

### Quality assessment

The authors critically evaluated the quality of the included articles. To do this, Joana Brigg’s Institute critical appraisal checklist for simple prevalence and an analytical cross-sectional study were used to assess the quality of the articles [24, 25]. Articles with a total quality score of more than 50% were labeled as paper-qualified articles, indicating a low risk of bias [25]. A discrepancy was observed between reviewers, and this was solved by discussion.

### Data synthesis and analysis

STATA software version 14 was used for systematic review and meta-analysis. For the systematic review, all eligible articles were summarized using a data extraction format, and then narration was done qualitatively. To assess the pooled prevalence of HPV vaccine uptake and its determinants (pooled effect size for each factor), a meta-analysis was conducted. Graphically, the Galbraith plot and the Forest plot were used. For the heterogeneity of the articles, statistically, I^2^ and Q^2^ statistics were used for heterogeneity assessment.

The heterogeneity I^2^ statistics values of 25, 50, and 75% were low, medium, and high heterogeneity, respectively. Additionally, heterogeneity was assessed using a random-effects model, subgroup analysis, and sensitivity analysis. A funnel plot and Egger’s regression tests were used for publication bias. The OR with a 95% CI and a *p*-value less than 0.05 was used to identify the determinants of HPV vaccine uptake in East Africa.

## Results

### Identification of the articles

**Fig. 1:**
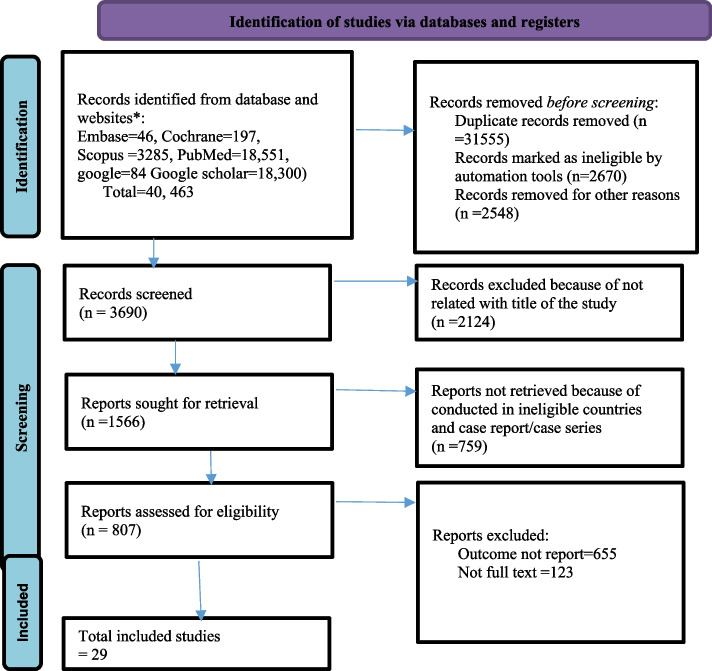
PRISMA flow diagram of study selection for pooled prevalence of HPV vaccine uptake and its determinants among females in East Africa, 2023

Both in electronic databases and on websites, 40,463 articles were identified. From all identified articles, 31,555 and 2670 were duplicated and ineligible articles, respectively. Moreover, a detailed and complete screening for selection was conducted among 3690 articles. After all, a total of 29 articles were selected for analysis (Fig 1).

### Characteristics of the included studies

Of the 29 included articles, the majority of 21 (72.4%) were published after COVID-19, and the included studies were conducted using a cross-sectional study design. The age of the participants included in the selected articles ranged from the minimum age of 9 years old to the maximum age of 24 years old, and the majority of the participants (82.8%) were adolescents.. Of the total selected articles, 10 (34.5%) were conducted in Uganda, followed by Ethiopia 7 (24.14%), and Kenya (5 (17.24%). Moreover, the minimum and maximum reported sample sizes in the included articles were 114 [26] and 687,500 [27], respectively **(**Table 2**).**

**Table 2:** characteristics of the included articles for HPV vaccine uptake among females in East Africa, 2023

S.No	Author	Publication year	age category of the participant	Country	Quality	Sample size	Prevalence
1	mekeds. S etal [26]	2022	adolescent	Ethiopia	low risk	660	59.39%
2	Mulugeta W/mariam Beyen1 etal [27]	2022	adolescent	Ethiopia	low risk	414	44.44%
3	Hareg Nigussie Kassa etal [28]	2021	adolescent	Ethiopia	low risk	591	66.50%
4	Mitiku Abera etal [29]	2023	adolescent	Ethiopia	low risk	423	52.01%
5	Etenesh Adela ethal [30]	2022	Youth	Ethiopia	low risk	633	16.11%
6	Hillary Mabeya etal [31]	2018	adolescent	Kenya	low risk	3083	63.80%
7	Tabitha C, Phylis J [32]	2023	adolescent	Kenya	low risk	35,195	17.00%
8	Tabitha C, Phylis J [32]	2023	adolescent	Kenya	low risk	32,254	15.00%
9	Christine Muthoni Karanja-Chege [25]	2022	adolescent	Kenya	low risk	687,500	16.00%
10	Hillary Mabeya etal [33]	2021	Youth	Kenya	low risk	300	9.33%
11	Lydia Patrick etal [34]	2022	adolescent	Uganda	low risk	288	69.79%
12	Esther Kisaakye etal [35]	2018	adolescent	Uganda	low risk	460	17.61%
13	Kelias Phiri Msyamboza etal [36]	2017	adolescent	Malawi	low risk	32,641	82.00%
14	Juliet Nabirye etal [37]	2020	adolescent	Uganda	low risk	407	14.00%
15	Alone Isabirye etal [38]	2022	adolescent	Uganda	low risk	6093	22.01%
16	Caroline Aruho etal [39]	2022	adolescent	Uganda	low risk	250	22.00%
17	Crystal N etal [25]	2013	adolescent	Somali	low risk	114	51.75%
18	Deborah Watson-Jones etal [40]	2012	adolescent	Tanzania	low risk	2180	76.10%
19	Edward Kumakech etal [41]	2017	Youth	Uganda	low risk	438	50.46%
20	Kakuru Glet Bitariho etal [42]	2022	adolescent	Uganda	low risk	524	8.59%
21	Mwansa Ketty Lubeya [43]	2023	adolescent	Zambia	low risk	400	53.75%
22	Ikrah Abdallah [44]	2021	adolescent	Tanzania	low risk	416	26.68%
23	Nchang’wa N * and Bruno S [45]	2022	adolescent	Tanzania	low risk	389	21.34%
24	Gerald Manasseh Kidogo [46]	2021	adolescent	Tanzania	low risk	450	24.67%
25	Catherine N and Banson JB [47]	2022	adolescent	Uganda	low risk	451	30.16%
26	Zaitune Nanyunja [48]	2019	adolescent	Uganda	low risk	380	9.21%
27	Andrew Kampikaho Turiho [49]	2015	adolescent	Uganda	low risk	385	22.60%
28	Tesfaye etal [50]	2017	Youth	Ethiopia	Low risk	267	15%
29	Terefe etal [51]	2022	Youth	Ethiopia	Low risk	568	42%

### Prevalence of HPV vaccine uptake in East Africa

**Fig. 2:**
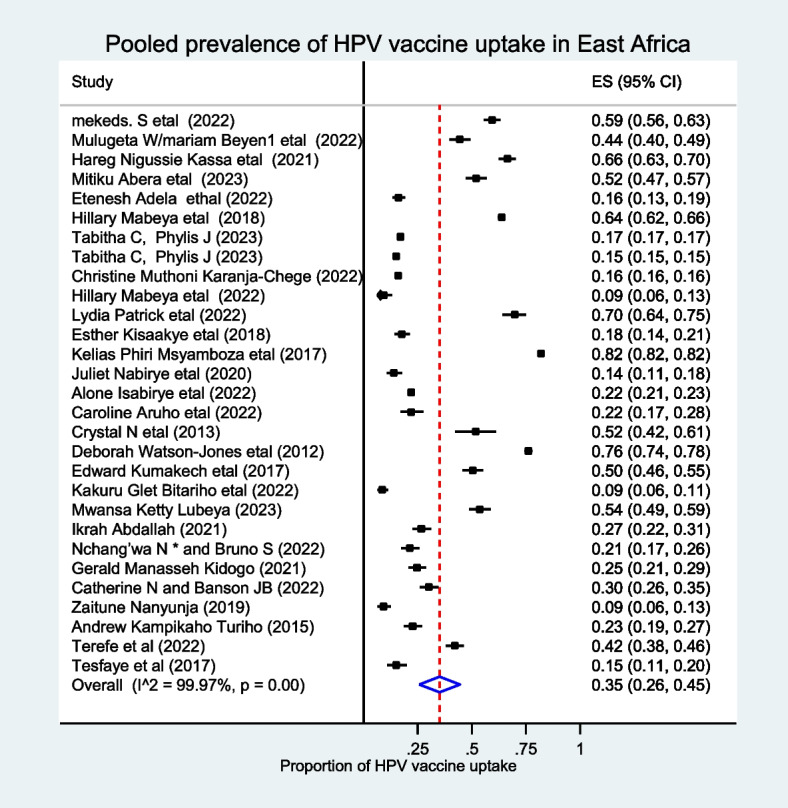
The pooled prevalence of HPV vaccine uptake among females in East Africa, 2023

The random effect model was used to analyze the pooled prevalence of HPV vaccine uptake, and thus the pooled prevalence of HPV vaccine uptake in East Africa was 35% (95% CI: 26–45%). Moreover, there was statistically significant heterogeneity between the studies (I^2^ = 99.97, *p*-value < 0.001) (Fig 2). As the evidence showed from Egger’s test and funnel plot, publication bias was not the problem of the current systematic review and meta-analysis (*P*-value = 0.135) (Supporting figure 1).

### Handling heterogeneity

**Fig. 3:**
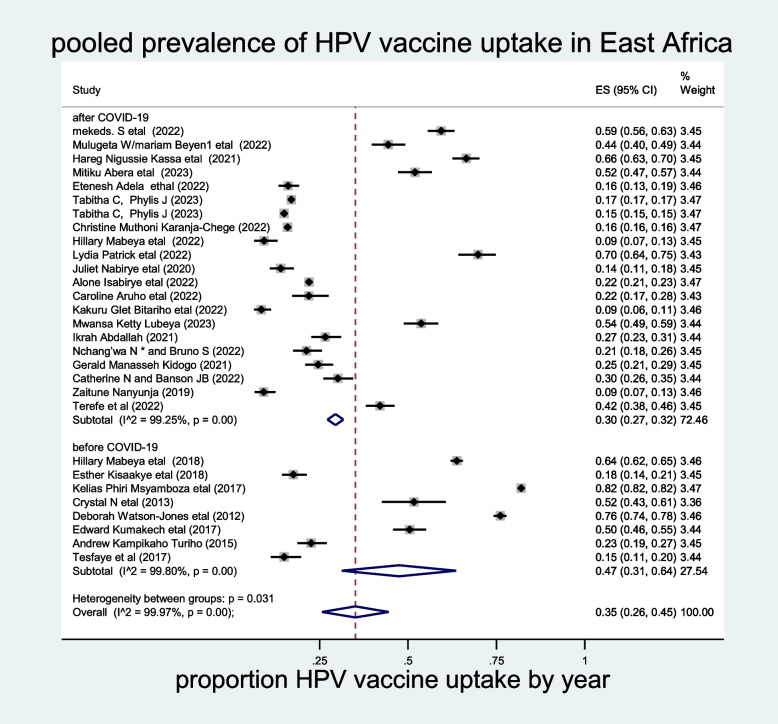
The prevalence of HPV vaccine uptake sub grouped by year of publication among females in East Africa, 2023

**Fig. 4:**
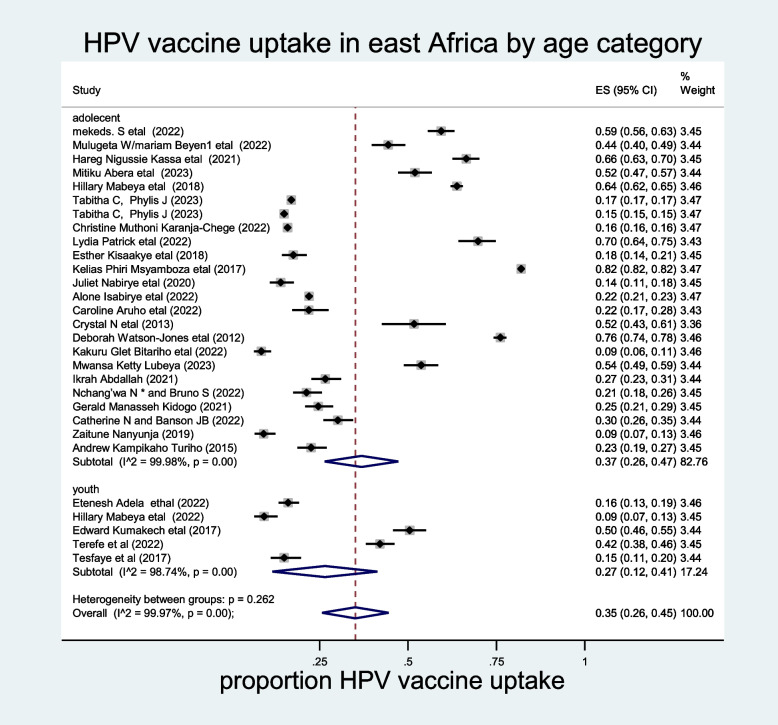
Sub group analysis of HPV vaccine uptake in east Africa by age category of the participants

The influence of a single study in meta-analysis estimation was evaluated using sensitivity analysis, and accordingly, the random effects model of sensitivity analysis showed that no study excessively influenced the overall pooled prevalence of HPV vaccine uptake in East Africa (Supporting figure 2). To resolve the heterogeneity across the studies, sub-group analysis by years of publication was also conducted. The pooled sub-group analysis of HPV vaccine uptake by years of publication was high among the studies conducted before the COVID-19 pandemic, which was 47% (95% CI: 31–64%) (Fig 3). Furthermore, a subgroup analysis by age category of the participant also revealed that the pooled prevalence of HPV vaccine uptake was higher among adolescents (37%; 95% CI: 26–47%) (Fig. 4).

### Factors associated with the uptake of HPV vaccine in East Africa

Using the random effect model, the pooled effect size was tried to be analyzed using variables such as knowledge about the HPV vaccine, attitude towards the HPV vaccine, residence, ever heard about the HPV vaccine, wealth index, mother’s education, father’s education, mother’s occupation, and family size. Finally, the factors that were significantly associated with the uptake of the HPV vaccine (*p*-value < 0.05) were knowledge about the HPV vaccine, attitude towards the HPV vaccine, ever heard about the HPV vaccine, residence, mother’s educational status, mother’s occupational status, wealth index, and family size. And thus, the meta-analysis of 10 studies conducted in Hawasa [28], Ambo [29], Minjar Ethiopia [30], Nekemit Ethiopia [31], Bahir Dar [32], Kenya [33], Lira district of Uganda [34], Kampala [35], Tanzania [36] and western Uganda [37] revealed that females who have good knowledge about HPV vaccine were 1.6 (OR = 1.6, 95% CI: 1.43–1.8, I^2^ = 90.5%, *p* = 0.001) times higher than their counterparts (Table 3, Supporting figure 3)**.** A meta-analysis of eight studies conducted in Hawasa [28], Ambo [29], Minjar Ethiopia [30], Nekemit Ethiopia [31], Bahir Dar [32], Lira district of Uganda [34], Kampala [35] and Tanzania [36] revealed that females who have a poor attitude towards the HPV vaccine were also 2.54 (OR = 2.54, 95% CI: 2.13–3.03, I^2^ = 70.8%, p = 0.001) times more likely to uptake the vaccine than their counterparts (Table 3, Supporting figure 4). A meta-analysis of three studies conducted in Hawasa [28], Tanzania [36] and Uganda [38] also reported that females who ever heard about the HPV vaccine were 1.41 (OR = 1.41, 95% CI: 1.03–1.94, I^2^ = 80.6%, *p* = 0.023) times more likely to uptake the HPV vaccine than their counterparts (Table 3, Supporting figure 5).

**Table 3:** Factors associated with the uptake of HPV vaccine among female in East Africa, 2023

Variable		OR(95%CI)	Heterogeneity (I^2^, *P*-value)	Total studies	Sample size
Knowledge	Good	1.6 (1.43, 1.8)*	90.5%, < 0.001	10	7562
	Poor	1	1		
Attitude about HPV vaccine	Positive	2.54(1.13, 3.03)*	70.8%, 0.001	8	4094
	Negative	1	1		
Residence	Urban	1.1 (1.01,1.2)*	94.1%, < 0.001	5	8150
	Rural	1	1		
Ever heard about HPV vaccine	Yes	1.41(1.03, 1.94)*	80.6%, 0.023	2	1120
	No	1	1		2846
Mother education	College and above	1.84 (1.03, 3.31)*	76.8%, 0.038	2	1083
	Primary education	1.26(0.77, 2.06)	0%, 0.371	2	1083
	No formal education	1	1		
Grade level	7–8	1.11 (0.75, 1.62)	0%, 0.34	2	1184
	4–6	1	1		
Family size	> = 9	0.79 (0,68, 0.98)*	65.6%, 0.088	2	6544
	< 9	1	1		
Wealth index	Middle	1.33 (1.04, 1.94)*	0%, 0.742	2	1049
	Poorest	1	1		
Mother occupation	Private employed	1.04(0.19, 5.78)	0%, 0.704	2	1184
	Government employed	1.18(0.12, 1.87)	85.2%, 0.009	2	1184
	Farmer	1	1		
Father educational status	Elementary	0.87(0.56, 1.34)	0%, 0.87	2	1083
	Secondary	1.11(0.6, 2.06)	35.1%, 0.215	2	1083
	college and above	1.49 (0.73,3.03)	0%, 0.462	2	1083
	No formal education	1	1		

* = significantly associated factors

Additionally, a meta-analysis of two studies conducted in Hawasa [28] and Nekemit Ethiopia [31] revealed that females whose mothers’ educational status above college was 1.84 (OR = 1.84, 95% CI: 1.03–3.31, I^2^ = 76.8%, *p* = 0.038) times higher to uptake the HPV vaccine than those without formal education (Table 3, Supporting figure 6).

Similarly, a meta-analysis of five studies conducted in Hawasa [28], Uganda [39], Nekemit Ethiopia [31], Tanzania [40] and Kampala [35] reported that females from urban areas were 1.1 (OR = 1.1, 95% CI: 1.01–1.2, I2 = 94.1%, *p* < 0.001) times more likely to uptake the HPV vaccine than rural residents (Table 3, Supporting figure 7). A meta-analysis of two studies conducted in Hawasa [28] and Tanzania [36] also reported that females from the middle wealth index family were 1.33 (OR = 1.33, 95% CI: 1.04–1.7, I^2^ = 0%, *p* = 0.742) times more likely to uptake the HPV vaccine than from the poorest wealth index family (Table 3, Supporting figure 8). Furthermore, a meta-analysis of two studies conducted in Uganda [39] and in the same country Buikwe district of Uganda [38] also revealed that the odds of HPV vaccine uptake among females whose family size was nine and above were reduced by 21% (OR = 0.79, 95% CI: 0.68–0.98, I2 = 65.6%, *p* = 0.088) than those whose family size was less than nine (Table 3, Supporting figure 9).

In the systematic review (qualitative narration), a study conducted in Hawasa [28] reported that the odds of HPV vaccine uptake in the availability of promotion were 2.53 (OR = 2.53, 95% CI: 1.51–4.26) times more likely than no availability of promotion [41]. A study in Uganda [34] also revealed that the availability of adequate vaccine and outreach vaccination practice were also 4.84 (OR = 4.84, 95% CI: 2.9–8.08) and 1.47 (OR = 1.47, 95% CI: 1.02–2.12) times more likely to uptake the HPV vaccine than their counterparts, respectively [34]. Furthermore, a study conducted in in Hawasa [28] also revealed that females who were supported by their family to receive the HPV vaccine were 4.3 (OR = 4.3, 95% CI: 2.98–6.21) times more likely to uptake the HPV vaccine than those without family support [41].

## Discussion

In the current systematic review and meta-analysis, an attempt has been made to assess the pooled prevalence of HPV vaccine uptake and its determinants in East Africa. Thus, the pooled prevalence of HPV vaccine uptake in East Africa was 35% (95% CI: 26–45%). The findings were consistent with a systematic and meta-analysis study conducted in Ethiopia’s (42.05%) [42]. But it was lower than the global strategic plan by 2030 (90%) [43], the global estimate of HPV vaccine coverage (77%) [44], a study conducted in 14 high-income countries among girls aged 13–19 years (83%) [45], in India (66%) [45], and in the United states of America (62.8%) [46]. The possible reason for this difference may be because of the variation in access to the HPV vaccine, the difference in socioeconomic status across the countries, the discrepancy in commitment to expand, and the difference in knowledge about the HPV vaccine. The other possible reason may be that the effects of COVID-19 significantly affect health services, including HPV vaccination practice. Similarly, in the current study, the lowest pooled prevalence of HPV vaccine uptake was reported after the COVID-19 pandemic. This implies that the COVID-19 pandemic strongly affects health service activities like health education about the HPV vaccine and the routine vaccination practice of the HPV vaccine. In contrast, the pooled prevalence of the HPV vaccine in East Africa was lower than in a study conducted in Sub-Saharan African countries (20%) [47]. This may be due to the difference in the study period. Because the level of awareness about the HPV vaccine and access to the vaccine can vary over time. Furthermore, a subgroup analysis by age category of the participant also revealed that the pooled prevalence of HPV vaccine uptake was higher among adolescents (37%; 95% CI: 26–47%). This might be associated with the fact that the majority of countries give attention and put an effort into the vaccination of adolescent girls. Countries have tried to expand school-based immunization campaigns among adolescents, which might be a possible justification for an increase in the adolescents’ vaccination rate.

Regarding the significant factors associated with the uptake of the HPV vaccine, having good knowledge about the HPV vaccine positively affected the uptake of the HPV vaccine. This finding was supported by a study of systematic and meta-analyses among teenagers [48], in Italy [49], in China [50] and Ethiopia [42]. This may be because knowledge is the key to taking an important prevention strategy. Knowledge about HPV infection and the HPV vaccine provides an important evidence-based decision for HPV vaccine uptake. Additionally, females who had ever heard about the HPV vaccine were more likely to uptake it, as supported by a study conducted in China and Malaysia [51, 52]. The possible explanation may be that females who have ever heard about the HPV vaccine could be the starting point for the uptake of the HPV vaccine, or it could provide an opportunity to have the most important messages about the benefit and the appropriate age of vaccination. In return, it could give them the confidence to take appropriate decisions.

Another factor that influences the uptake of the HPV vaccine is the attitude towards the HPV vaccine. Thus, females who had a positive attitude towards the HPV vaccine were more likely to uptake the HPV vaccine than those who had a negative attitude towards the HPV vaccine. This finding was supported by a study conducted using systematic and meta-analyses [42, 48]. This might be associated with the fact that a female’s healthy lifestyle can be influenced by their attitude. The most discouraging factor of female practice is generated by their negative attitude. Females reacting negatively to a stimulus about the HPV vaccine may reduce their motivation to take the vaccine. Another piece of evidence also showed that a negative attitude is the most common barrier to the uptake of the HPV vaccine [53].

The residence was also a significant factor in the uptake of the HPV vaccine. Females who were from urban residences were more likely to uptake the HPV vaccine than rural residents. This was supported by a systematic and meta-analytic study conducted in Ethiopia [42]. This could be justified by the fact that female urban residents are more likely to access health information and health services than rural residents. Additionally, females whose mothers’ educational status was above college were more likely to uptake the HPV vaccine than those without formal education. This was supported by a study conducted in China [54]. This could be justified by the fact that educated mothers have more information, knowledge, and socioeconomic status than uneducated mothers, which enables them to advise and encourage their children to uptake the HPV vaccine.

Females from the middle wealth index family were more likely to uptake the HPV vaccine than from the poorest wealth index family. This was supported by a study conducted in the United States of America (USA) [55]. This may be because economic status is an important input (enabling factor) for the uptake of health services, better information access, and a healthier lifestyle. The other possible justification is that socioeconomic status can provide and encourage positive health behavior [39].

Family size was also an important factor in the uptake of the HPV vaccine. Females whose family size was nine and above were less likely to uptake the HPV vaccine than those whose family size was less than nine. This finding was agreed upon by a study conducted in Nigeria [56]. This is because large family sizes negatively affect the utilization of health services [56].

In the systematic review (qualitative narration), the availability of vaccines at the site of vaccination was a positive predictor of HPV vaccine uptake [34]. This finding was agreed upon by a study conducted in South Michigan [57]. This is because adequate availability of vaccine at the site of vaccination improves the vaccine uptake from 11% before availability to 16% after availability [34]. Even if the availability of vaccines at the site of vaccination is crucial for the uptake of the HPV vaccine, in itself, it is not enough for the sustainable improvement of HPV vaccination coverage. It also needs adequate resources to support the program, health education and counselling services, health professional capacity building, proper or fair distribution of the vaccine, community outreach vaccinations, and the availability of promotion about the HPV vaccine. Furthermore, females who had family or guardian support to receive the HPV vaccine were more likely to uptake the HPV vaccine than those who had no family or guardian support [41].. This was supported by a study conducted in the USA and Victoria [58, 59]. This could be because those who are supported by their family or guardian can get adequate knowledge about the HPV vaccine, have a favorable attitude towards HPV vaccine uptake, and can provide an important step in deciding the uptake of the HPV vaccine. Additionally, most of the time, adolescents’ decisions rest on their family or guardian, and thus family support offers a crucial factor in expanding vaccination practice. The limitation of the current study was that articles without full text were excluded, which may affect the actual pooled effect.

## Conclusion

As compared to the global strategic plan, the pooled prevalence of HPV vaccine uptake in east Africa was low. The uptake of HPV vaccine was higher among adolescents than youths. Knowledge about the HPV vaccine, attitude towards the HPV vaccine, ever hearing about the HPV vaccine, residence, mother’s educational status, mother’s occupational status, wealth index, and family size were the significant determinants of HPV vaccine uptake. Therefore, we recommend focusing on awareness creation and behavioral change to expand the uptake of vaccines in East Africa.

### Supplementary Information

**Supplementary Material 1.**

**Supplementary Material 2.**

**Supplementary Material 3.**

**Supplementary Material 4.**

**Supplementary Material 5.**

**Supplementary Material 6.**

**Supplementary Material 7.**

**Supplementary Material 8.**

**Supplementary Material 9.**

## Data Availability

All the data and supporting files are in the articles.

## Abbreviations

COVID-19: Corona Virus Disease
CI: Confidence Interval
HPV: Human Papillomavirus
OR: Odds Ratio
PRISMA: Preferred Reporting Item for Systematic Review and Meta-Analysis
USA: United State of America
