# Supplementary figures and images for: Uptake of human papilloma virus vaccine and its determinants among females in East Africa: a systematic review and meta-analysis
Source: BMC Public Health. 2024 Mar 18;24:842. doi: 10.1186/s12889-024-18141-5 (PMC10949808; doi:10.1186/s12889-024-18141-5)

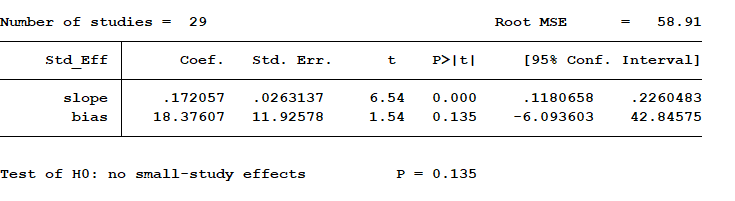


**Supporting figure-1: funnel plot of HPV vaccine uptake among females in East Africa, 2023.**

Supplement: Supplementary file 1 — Supplementary Material 1. [file 12889_2024_18141_MOESM1_ESM.docx]
