# Supplementary material for: Uptake of human papilloma virus vaccine and its determinants among females in East Africa: a systematic review and meta-analysis
Source: BMC Public Health. 2024 Mar 18;24:842. doi: 10.1186/s12889-024-18141-5 (PMC10949808; doi:10.1186/s12889-024-18141-5)
Supplement: Supplementary file 5 — Supplementary Material 5. [file 12889_2024_18141_MOESM5_ESM.docx]

**Supporting figure-5: effect of ever heard about HPV vaccine on HPV vaccine uptake among females in East Africa, 2023.**
